# Supplementary material for: Screening for Virulence-Related Genes via a Transposon Mutant Library of Streptococcus suis Serotype 2 Using a Galleria mellonella Larvae Infection Model
Source: Microorganisms. 2022 Apr 21;10(5):868. doi: 10.3390/microorganisms10050868 (PMC9143085; doi:10.3390/microorganisms10050868)
Supplement: Supplementary file 1 [file microorganisms-10-00868-s001.zip › Figure S1.pdf]

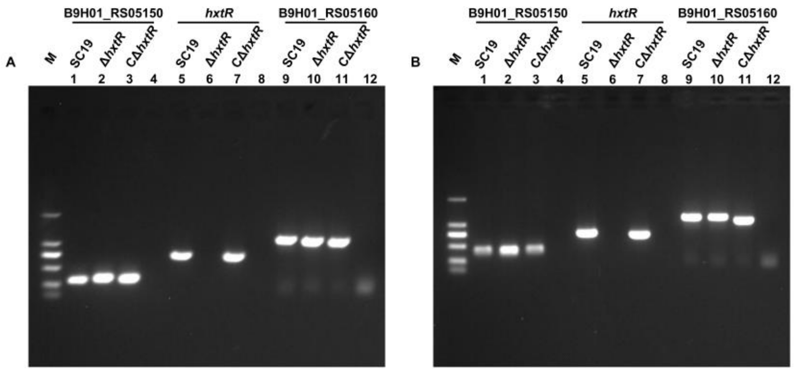

Figure S1. Verification of Δ*hxtR* and CΔ*hxtR* by PCR and RT-PCR. (A) Identification of Δ*hxtR* and CΔ*hxtR* by PCR. Lanes 1-4 show the amplification of the upstream gene of *hxtR* using the primer pair 5150-F/R. Lanes 5-8 show the amplification of *hxtR* using the primer pair *hxtR*-F/R. Lanes 9-12 show the amplification of the downstream gene of *hxtR* using the primer pair 5160-F/R. In lanes 1, 5, and 9, the genomic DNA of *S. suis* SC19 was used as the template for PCR. In lanes 2, 6, and 10, the genomic DNA of Δ*hxtR* was used as the template for PCR. In lanes 3, 7, and 11, the genomic DNA of CΔ*hxtR* was used as the template for PCR. Lanes 4, 8, and 12 represent the negative control. (B) Identification of Δ*hxtR* and CΔ*hxtR* by RT-PCR. Same primers were used as above and the cDNA of SC19, Δ*hxtR*, and CΔ*hxtR* were used as templates in the PCR.
