# Supplementary material for: Screening for Virulence-Related Genes via a Transposon Mutant Library of Streptococcus suis Serotype 2 Using a Galleria mellonella Larvae Infection Model
Source: Microorganisms. 2022 Apr 21;10(5):868. doi: 10.3390/microorganisms10050868 (PMC9143085; doi:10.3390/microorganisms10050868)
Supplement: Supplementary file 1 [file microorganisms-10-00868-s001.zip › Table S1.pdf]

Table S1. Tn mutants information.

Locus Tag	Protein Name	Tn Mutants
B9H01_RS05050	ABC transporter ATP-binding protein	Tn52, Tn64, Tn123, Tn180, Tn204, Tn216, Tn393, Tn483, Tn502, Tn562, Tn578, Tn672, Tn679, Tn699, Tn746, Tn1012, Tn1025, Tn1030, Tn1031, Tn1050, Tn1074, Tn1093, Tn1095, Tn1145, Tn1171, Tn1219, Tn1238, Tn1243, Tn1368, Tn1370, Tn1553, Tn1566, Tn1609, Tn1620, Tn1660, Tn1666, Tn1679, Tn1768, Tn1871, Tn1876
B9H01_RS05005	8-oxo-dgtp diphosphatase	Tn94, Tn136, Tn141, Tn199, Tn203, Tn214, Tn221, Tn265, Tn270, Tn327, Tn406, Tn422, Tn430, Tn487, Tn570, Tn771, Tn785, Tn786, Tn809, Tn818, Tn864, Tn871, Tn915, Tn917, Tn976, Tn977, Tn1183, Tn1199, Tn1473, Tn1552, Tn1595, Tn1614, Tn1667, Tn1671, Tn1765, Tn1830
B9H01_RS05070	Protein (LacX)	Tn119, Tn177, Tn207, Tn305, Tn428, Tn436, Tn479, Tn493, Tn521, Tn696, Tn726, Tn762, Tn789, Tn844, Tn859, Tn867, Tn886, Tn920, Tn964, Tn1072, Tn1106, Tn1178, Tn1280, Tn1286, Tn1357, Tn1379, Tn1388, Tn1565, Tn1649, Tn1695, Tn1772, Tn1826, Tn1840
B9H01_RS04990	1,4-alpha-glucan branching protein (GlgB)	Tn82, Tn120, Tn174, Tn205, Tn206, Tn253, Tn621, Tn695, Tn703, Tn766, Tn842, Tn866, Tn882, Tn1027, Tn1215, Tn1236, Tn1269, Tn1325, Tn1390, Tn1423, Tn1444, Tn1457, Tn1723, Tn1790, Tn1892, Tn1902, Tn1909
B9H01_RS05230	Peptidase	Tn2, Tn269, Tn273, Tn306, Tn381, Tn398, Tn449, Tn509, Tn654, Tn727, Tn739, Tn787, Tn887, Tn940, Tn952, Tn958, Tn989, Tn998, Tn1060, Tn1320, Tn1509, Tn1663, Tn1744
B9H01_RS04750	Hypothetical protein	Tn9, Tn46, Tn93, Tn329, Tn468, Tn730, Tn742, Tn926, Tn943, Tn1043, Tn1115, Tn1160, Tn1256, Tn1265, Tn1323, Tn1342, Tn1431, Tn1451, Tn1471, Tn1617
B9H01_RS05235	JAB domain-containing protein	Tn13, Tn36, Tn102, Tn226, Tn233, Tn626, Tn653, Tn682, Tn769, Tn799, Tn833, Tn985, Tn1127, Tn1278, Tn1297, Tn1469, Tn1562, Tn1714, Tn1721, Tn1752
B9H01_RS04765	Glucan-binding protein	Tn15, Tn92, Tn191, Tn290, Tn303, Tn457, Tn477, Tn942, Tn996, Tn1179, Tn1214, Tn1331, Tn1391, Tn1420, Tn1540, Tn1672, Tn1687, Tn1889

Locus Tag	Protein Name	Tn Mutants
B9H01_RS03005	Hypothetical protein	Tn38, Tn166, Tn227, Tn399, Tn563, Tn591, Tn592, Tn645, Tn733, Tn961, Tn983, Tn1053, Tn1133, Tn1389, Tn1422, Tn1593, Tn1624
B9H01_RS04780	CHAP domain-containing protein	Tn66, Tn95, Tn353, Tm488, Tn863, Tn1105, Tn1114, Tn1130, Tn1142, Tn1275, Tn1277, Tn1338, Tn1371, Tn1812, Tn1820, Tn1870, Tn1914
B9H01_RS05090	Transcriptional anti-terminator	Tn40, Tn44, Tn301, Tn313, Tn564, Tn567, Tn670, Tn671, Tn717, Tn776, Tn855, Tn931, Tn1049, Tn1456, Tn1682, Tn1766
B9H01_RS02990	MULTISPECIES: hypothetical protein	Tn272, Tn279, Tn362, Tn752, Tn795, Tn807, Tn810, Tn1067, Tn1148, Tn1245, Tn1454, Tn1574, Tn1722
B9H01_RS05000	Amino acid ABC transporter substrate-binding protein	Tn105, Tn263, Tn345, Tn403, Tn919, Tn1042, Tn1107, Tn1482, Tn1492, Tn1705, Tn1720, Tn1801, Tn1852
B9H01_RS04475	Asparagine synthetase	Tn33, Tn55, Tn87, Tn112, Tn262, Tn264, Tn623, Tn690, Tn714, Tn824, Tn881, Tn1777
B9H01_RS04805	Conjugal transfer protein (TraG)	Tn32, Tn104, Tn172, Tn188, Tn317, Tn318, Tn387, Tn451, Tn768, Tn1120, Tn1458, Tn1895
B9H01_RS05075	6-phospho-beta-galactosidase	Tn460, Tn597, Tn784, Tn792, Tn880, Tn897, Tn1164, Tn1249, Tn1362, Tn1403, Tn1732, Tn1874
B9H01_RS05065	Hypothetical protein	Tn47, Tn126, Tn504, Tn662, Tn979, Tn993, Tn1318, Tn1696, Tn1729, Tn1747, Tn1884
B9H01_RS04330	Phosphomannomutase/phosphoglucomutase	Tn4, Tn73, Tn417, Tn712, Tn750, Tn1233, Tn1255, Tn1260, Tn1594, Tn1731
B9H01_RS04675	ABC transporter	Tn938, Tn1161, Tn1163, Tn1460, Tn1572, Tn1691, Tn1796, Tn1811, Tn1861, Tn1897
B9H01_RS04775	Nucleotidyl transferase abieii/abigii toxin family protein	Tn74, Tn245, Tn250, Tn605, Tn648, Tn1202, Tn1483, Tn1501, Tn1564, Tn1899
B9H01_RS04995	Amino acid ABC transporter substrate-binding protein	Tn200, Tn201, Tn431, Tn556, Tn763, Tn1094, Tn1190, Tn1428, Tn1701, Tn1873
B9H01_RS05085	MULTISPECIES: PTS lactose/cellobiose transporter subunit IIA	Tn348, Tn584, Tn665, Tn738, Tn910, Tn928, Tn947, Tn1155, Tn1167, Tn1725
B9H01_RS05160	A/G-specific adenine glycosylase	Tn299.Tn409, Tn734, Tn909, Tn949, Tn1271, Tn1316, Tn1401, Tn1819, Tn1823
B9H01_RS00700	Cell division protein (FtsK)	Tn284, Tn357, Tn529, Tn802, Tn814, Tn1057, Tn1226, Tn1683, Tn1726

Locus Tag	Protein Name	Tn Mutants
B9H01_RS03155	N-acetyl-beta-hexosaminidase	Tn684, Tn890, Tn1041, Tn1128, Tn1151, Tn1192, Tn1495, Tn1760, Tn1894
B9H01_RS04355	ABC transporter permease	Tn7, Tn56, Tn351, Tn508, Tn1103, Tn1169, Tn1445, Tn1449, Tn1741
B9H01_RS05060	MULTISPECIES: hypothetical protein	Tn453, Tn523, Tn634, Tn728, Tn788, Tn877, Tn885, Tn1396, Tn1644
B9H01_RS05145	Hypothetical protein	Tn29, Tn211, Tn354, Tn835, Tn902, Tn1311, Tn1497, Tn1527, Tn1785
B9H01_RS04785	Atpase AAA	Tn127, Tn601, Tn1110, Tn1217, Tn1272, Tn1340, Tn1619, Tn1709
B9H01_RS05015	Peptidase M26	Tn249, Tn914, Tn1292, Tn1742, Tn1783, Tn1817, Tn1821, Tn1855
B9H01_RS05505	Hypothetical protein	Tn238, Tn246, Tn1146, Tn1289, Tn1398, Tn1546, Tn1605, Tn1681
B9H01_RS04375	MULTISPECIES: ABC transporter ATP-binding protein	Tn729, Tn732, Tn791, Tn837, Tn1073, Tn1673, Tn1887
B9H01_RS04385	Hypothetical protein	Tn526, Tn843, Tn1017, Tn1248, Tn1670, Tn1805, Tn1911
B9H01_RS04405	Hypothetical protein	Tn122, Tn1121, Tn1287, Tn1315, Tn1502, Tn1903, Tn1905
B9H01_RS04450	ABC transporter permease	Tn23, Tn86, Tn169, Tn850, Tn944, Tn1531
B9H01_RS04570	MULTISPECIES: antirestriction protein (ArdA)	Tn170, Tn1054, Tn1138, Tn1375, Tn1459, Tn1841
B9H01_RS04610	ATP-dependent endonuclease	Tn164, Tn1147, Tn1177, Tn1349, Tn1384, Tn1486
B9H01_RS05095	Tagatose-bisphosphate aldolase	Tn168, Tn501, Tn805, Tn1013, Tn1281, Tn1762
B9H01_RS05105	MULTISPECIES: galactose-6-phosphate isomerase subunit (LacB)	Tn183, Tn314, Tn613, Tn649, Tn823, Tn1435
B9H01_RS05125	Hypothetical protein	Tn54, Tn235, Tn861, Tn995, Tn1065, Tn1208
B9H01_RS05485	Permease	Tn81, Tn242, Tn820, Tn899, Tn1036, Tn1776
B9H01_RS05570	VTC domain-containing protein	Tn1038, Tn1186, Tn1230, Tn1418, Tn1462, Tn1868
B9H01_RS01170	DUF4838 domain-containing protein	Tn78, Tn239, Tn254, Tn1327, Tn1567
B9H01_RS01945	ATP-binding protein	Tn500, Tn1019, Tn1476, Tn1571, Tn1745
B9H01_RS03545	Glycerol dehydrogenase	Tn1076, Tn1306, Tn1563, Tn1757, Tn1857
B9H01_RS04365	ABC transporter permease	Tn1170, Tn1242, Tn1414, Tn1446, Tn1716

Locus Tag	Protein Name	Tn Mutants
B9H01_RS04465	ABC transporter ATP-binding protein	Tn352, Tn359, Tn1298, Tn1425, Tn1906
B9H01_RS04920	Septum formation inhibitor (Maf)	Tn1227, Tn1479, Tn1650, Tn1703, Tn1715
B9H01_RS05055	O-acetylhomoserine aminocarboxypropyltransferase/cysteine synthase	Tn294, Tn402, Tn439, Tn841, Tn1328
B9H01_RS05140	Integrase	Tn34, Tn309, Tn311, Tn312, Tn583
B9H01_RS07430	Bifunctional metallophosphatase/5\'-nucleotidase	Tn27, Tn61, Tn986, Tn1189, Tn1372
B9H01_RS01055	PTS sugar transporter subunit IIC	Tn736, Tn1075, Tn1541, Tn1918
B9H01_RS01165	PTS sugar transporter subunit IIC	Tn713, Tn1048, Tn1218, Tn1426
B9H01_RS01940	Hypothetical protein	Tn222, Tn956, Tn1089, Tn1603
B9H01_RS03550	Glycerol dehydrogenase	Tn63, Tn282, Tn471, Tn1771
B9H01_RS03990	Subtilisin-like serine protease(SspA)	Tn98, Tn371, Tn1099, Tn1283
B9H01_RS04555	MULTISPECIES: membrane protein	Tn689, Tn819, Tn1339, Tn1622
B9H01_RS04560	MULTISPECIES: ATP/GTP-binding protein	Tn498, Tn518, Tn673, Tn1141
B9H01_RS04650	DNA-binding response regulator	Tn528, Tn538, Tn542, Tn1263
B9H01_RS04695	MULTISPECIES: recombinase family protein	Tn42， Tn425， Tn1382， Tn1788
B9H01_RS04740	Hypothetical protein	Tn3， Tn274， Tn1815， Tn1816
B9H01_RS04795	Conjugal transfer protein (TrbL)	Tn369, Tn610, Tn1098, Tn1480
B9H01_RS04815	CPBP family intramembrane metalloprotease	Tn67, Tn397, Tn1558, Tn1698
B9H01_RS04885	Coa-disulfide reductase	Tn148, Tn646, Tn1229, Tn1310
B9H01_RS05120	Deor/glpr transcriptional regulator	Tn396, Tn506, Tn1001, Tn1773
B9H01_RS05130	DNA-binding protein	Tn219, Tn987, Tn1033, Tn1252
B9H01_RS05155	XRE family transcriptional regulator	Tn154, Tn589, Tn898, Tn1864
B9H01_RS05170	ABC transporter ATP-binding protein	Tn537, Tn554, Tn1174, Tn1913
B9H01_RS05640	Beta-glucuronidase	Tn271, Tn822, Tn1285, Tn1369
B9H01_RS05650	Glucuronide permease	Tn116, Tn632, Tn663, Tn1188

Locus Tag	Protein Name	Tn Mutants
B9H01_RS05870	Hyaluronidase	Tn660, Tn700, Tn813, Tn980
B9H01_RS05885	PTS mannose/fructose/sorbose /N-acetylgalactosamine transporter subunit IIC	Tn125, Tn704, Tn705, Tn969
B9H01_RS05905	Bifunctional 2-keto-4-hydroxyglutarate aldolase/2-keto-3-deoxy-6-phosphogluconate aldolase	Tn132, Tn1587, Tn1750, Tn1882
B9H01_RS05920	Gluconate 5-dehydrogenase	Tn622, Tn702, Tn1015, Tn1392
B9H01_RS06130	Histidine triad protein	Tn656, Tn816, Tn941, Tn1775
B9H01_RS09215	Alpha-mannosidase	Tn41, Tn404, Tn701, Tn1610
B9H01_RS09610	Radical SAM protein	Tn19, Tn851, Tn916, Tn1253
B9H01_RS00850	Bifunctional folylpolyglutamate synthase/dihydrofolate synthase	Tn698, Tn1092, Tn1781
B9H01_RS01175	Sugar ABC transporter substrate-binding protein	Tn778, Tn1573, Tn1810
B9H01_RS01835	Deoxyguanosinetriphosp hate triphosphohydrolase	Tn666, Tn1150, Tn1464
B9H01_RS01860	Copper homeostasis protein (CutC)	Tn187, Tn337, Tn338
B9H01_RS02280	Beta-galactosidase	Tn146, Tn455, Tn1168
B9H01_RS02335	Hypothetical protein	Tn599, Tn963, Tn1544
B9H01_RS02800	Bifunctional dnaq family exonuclease/ATP-dependent helicase	Tn854, Tn1232, Tn1706
B9H01_RS03230	MULTISPECIES: rrna pseudouridine synthase	Tn296, Tn959, Tn990
B9H01_RS03235	Glutathione peroxidase	Tn1158, Tn1554, Tn1843
B9H01_RS03345	Cation transporter	Tn412, Tn1692, Tn1727
B9H01_RS03415	DEAD/DEAH box helicase	Tn17, Tn1135, Tn1493
B9H01_RS04305	DUF3307 domain-containing protein	Tn486, Tn1237, Tn1608
B9H01_RS04390	Carbamoyl phosphate synthase large subunit	Tn1351, Tn1604, Tn1616
B9H01_RS04425	Site-specific integrase	Tn497, Tn1003, Tn1500
B9H01_RS04430	MULTISPECIES: DUF3173 domain-containing protein	Tn1394, Tn1601, Tn1748
B9H01_RS04440	Sensor histidine kinase	Tn1172, Tn1268, Tn1294

Locus Tag	Protein Name	Tn Mutants
B9H01_RS04445	DNA-binding response regulator	Tn491, Tn527, Tn1519
B9H01_RS04480	Chromosome segregation atpase	Tn1485, Tn1506, Tn1640
B9H01_RS04495	ATP-binding protein	Tn96, Tn295, Tn1119
B9H01_RS04640	DNA primase	Tn1212, Tn1258, Tn1319
B9H01_RS04680	Lantibiotic-modifying protein	Tn1470，Tn1664，Tn1809
B9H01_RS04705	MULTISPECIES: hypothetical protein	Tn118，Tn873，Tn1353
B9H01_RS04755	Hypothetical protein	Tn492, Tn745, Tn1499
B9H01_RS04810	Hypothetical protein	Tn561, Tn572, Tn675
B9H01_RS04840	Hypothetical protein	Tn62, Tn524, Tn1665
B9H01_RS04865	DNA-protecting protein (DprA)	Tn30, Tn1367, Tn1838
B9H01_RS05010	N-acetyltransferase	Tn530, Tn1046, Tn1159
B9H01_RS05045	N-acetyltransferase	Tn8, Tn389, Tn546
B9H01_RS05080	PTS lactose transporter subunit IIBC	Tn853, Tn1132, Tn1885
B9H01_RS05110	Galactose-6-phosphate isomerase subunit (LacA)	Tn101, Tn1347, Tn1397
B9H01_RS05525	Membrane protein	Tn937, Tn1393, Tn1538
B9H01_RS05600	Glycosyl hydrolase	Tn711, Tn997, Tn1241
B9H01_RS05620	Mannonate dehydratase	Tn370, Tn968, Tn1317
B9H01_RS05645	Sugar kinase	Tn489, Tn503, Tn507
B9H01_RS06510	Atpase	Tn333, Tn336, Tn1759
B9H01_RS07195	Transcription anti-terminator (BglG)	Tn108, Tn111, Tn1140
B9H01_RS09370	Glsb/yeaq/ymge family stress response membrane protein	Tn231, Tn275, Tn1551
B9H01_RS00720	DUF2971 domain-containing protein	Tn308, Tn1549
B9H01_RS00815	Type II secretion system protein	Tn466, Tn467
B9H01_RS00830	Hypothetical protein	Tn37, Tn423
B9H01_RS00935	Aromatic acid exporter family protein	Tn1615, Tn1677
B9H01_RS00980	Sugar ABC transporter substrate-binding protein	Tn445, Tn494
B9H01_RS01000	MULTISPECIES: branched-chain amino acid ABC transporter permease	Tn755, Tn1157

Locus Tag	Protein Name	Tn Mutants
B9H01_RS01015	Hypothetical protein	Tn1312, Tn838
B9H01_RS01035	ABC transporter ATP-binding protein	Tn948, Tn1474
B9H01_RS01080	Glycerophosphoryl diester phosphodiesterase	Tn889, Tn1290
B9H01_RS01370	Tetr/acrr family transcriptional regulator	Tn1066, Tn1807
B9H01_RS01465	Bifunctional acetaldehyde-coa/alcohol dehydrogenase	Tn1047, Tn1321
B9H01_RS01475	ABC transporter ATP-binding protein	Tn541, Tn1173
B9H01_RS01575	MULTISPECIES: lipoate--protein ligase family protein	Tn761, Tn1024
B9H01_RS01600	Hypothetical protein	Tn212, Tn590
B9H01_RS01965	ATP-dependent Clp protease ATP-binding subunit	Tn972, Tn1794
B9H01_RS02170	Helicase	Tn391, Tn1337
B9H01_RS02475	DUF1827 domain-containing protein	Tn531, Tn1774
B9H01_RS02480	NUDIX hydrolase	Tn1029, Tn1117
B9H01_RS03000	Hypothetical protein	Tn88, Tn966
B9H01_RS03250	Ferrichrome ABC transporter substrate-binding protein	Tn65, Tn1326
B9H01_RS03320	Phosphoserine aminotransferase	Tn1329, Tn1522
B9H01_RS03365	DUF975 domain-containing protein	Tn868, Tn874
B9H01_RS03375	DUF975 domain-containing protein	Tn496, Tn1662
B9H01_RS03530	PTS sugar transporter subunit IIC	Tn255, Tn464
B9H01_RS03535	Formate C-acetyltransferase/glycerol dehydratase family glycyl radical enzyme	Tn793, Tn1559
B9H01_RS03600	Oligoendopeptidase F	Tn49, Tn1250
B9H01_RS03650	ECF transporter S component	Tn342, Tn1343
B9H01_RS04135	Calcium-binding protein	Tn297, Tn1835, Tn1847
B9H01_RS04200	Hypothetical protein	Tn35, Tn731, Tn1335

Locus Tag	Protein Name	Tn Mutants
B9H01_RS04245	Hypothetical protein	Tn515, Tn1282
B9H01_RS04260	Hypothetical protein	Tn743, Tn744
B9H01_RS04275	Hypothetical protein	Tn179, Tn287
B9H01_RS04280	Glutamine-hydrolyzing GMP synthase	Tn664, Tn667
B9H01_RS04290	Putative DNA-binding protein	Tn1599, Tn1600
B9H01_RS04325	Tyrosine recombinase (XerS)	Tn1646, Tn1707
B9H01_RS04340	DNA-binding response regulator	Tn565, Tn1655
B9H01_RS04370	ABC transporter permease	Tn1204, Tn1734
B9H01_RS04380	Lipoprotein	Tn692, Tn1769
B9H01_RS04400	Esterase	Tn53, Tn470
B9H01_RS04420	HAD family hydrolase	Tn11, Tn930
B9H01_RS04525	MULTISPECIES: XRE family transcriptional regulator	Tn1355, Tn1407
B9H01_RS04535	MULTISPECIES: tetracycline resistance ribosomal protection protein (Tet(M))	Tn79, Tn1570
B9H01_RS04545	MULTISPECIES: conjugal transfer protein	Tn247, Tn971
B9H01_RS04580	MULTISPECIES: XRE family transcriptional regulator	Tn697, Tn1154
B9H01_RS04590	MULTISPECIES: DNA translocase (FtsK)	Tn71, Tn1026
B9H01_RS04605	ATP-dependent helicase	Tn588, Tn1824
B9H01_RS04645	Hypothetical protein	Tn869, Tn1293
B9H01_RS04655	ATP-binding protein	Tn443, Tn1648
B9H01_RS04715	MULTISPECIES: aminoglycoside nucleotidyltransferase ANT(6)-Ia	Tn236，Tn410
B9H01_RS04730	DUF4135 domain-containing protein	Tn540，Tn1642
B9H01_RS04830	MULTISPECIES: hypothetical protein	Tn1193, Tn1879
B9H01_RS04880	Rhodanese-like domain-containing protein	Tn630, Tn1917
B9H01_RS04895	Hemolysin III	Tn950, Tn951

Locus Tag	Protein Name	Tn Mutants
B9H01_RS04900	Ydcf family protein	Tn1087, Tn1792
B9H01_RS05135	Integrase	Tn316, Tn1406
B9H01_RS05150	Hypothetical protein	Tn936, Tn1591
B9H01_RS05335	DNA-binding response regulator	Tn481, Tn716
B9H01_RS05480	Hypothetical protein	Tn852, Tn1886
B9H01_RS05495	Hypothetical protein	Tn1472, Tn1694
B9H01_RS05500	DUF1700 domain-containing protein	Tn165, Tn510
B9H01_RS05530	Cation transporter	Tn106, Tn1405
B9H01_RS05565	DUF4956 domain-containing protein	Tn1004, Tn1718
B9H01_RS05605	Beta-hexosamidase	Tn454, Tn560
B9H01_RS05610	HAD family hydrolase	Tn495, Tn604
B9H01_RS05615	MULTISPECIES: dioxygenase	Tn153, Tn544
B9H01_RS05625	Glucuronate isomerase	Tn186, Tn1753
B9H01_RS05740	DNA alkylation repair protein	Tn244, Tn1689
B9H01_RS05815	Citrate synthase	Tn815, Tn834
B9H01_RS05895	Glucuronyl hydrolase	Tn375, Tn694
B9H01_RS06065	ABC transporter permease	Tn1380, Tn1653
B9H01_RS06070	MULTISPECIES: lipoprotein	Tn160, Tn1437
B9H01_RS06460	ABC transporter substrate-binding protein	Tn893, Tn1833
B9H01_RS06805	Ferrous iron transport protein B	Tn1240, Tn1786
B9H01_RS07165	Prolyl-trna synthetase associated domain-containing protein	Tn325, Tn558
B9H01_RS07380	Hypothetical protein	Tn58, Tn811
B9H01_RS07495	Alpha-glucosidase	Tn708, Tn720
B9H01_RS07595	Hypothetical protein	Tn356, Tn368
B9H01_RS07690	ABC transporter permease	Tn571, Tn1529
B9H01_RS07960	DUF2140 domain-containing protein	Tn224, Tn1883
B9H01_RS08070	Prepilin peptidase	Tn1079, Tn1728
B9H01_RS08250	Aminopeptidase C	Tn796, Tn808
B9H01_RS08490	MATE family efflux transporter	Tn461, Tn1090

Locus Tag	Protein Name	Tn Mutants
B9H01_RS08600	Restriction endonuclease subunit S	Tn878, Tn1247
B9H01_RS08855	Membrane protein	Tn514, Tn932
B9H01_RS09320	Rrna pseudouridine synthase	Tn113, Tn586
B9H01_RS09530	Serine protease	Tn1630, Tn1737
B9H01_RS09560	Rlua family pseudouridine synthase	Tn1058, Tn1919
B9H01_RS10245	Hypothetical protein	Tn407, Tn424
B9H01_RS10350	Thiol reductase thioredoxin	Tn499, Tn522
B9H01_RS00230	MULTISPECIES: phosphoribosyl aminoimidazolesuccino carboxamide synthase	Tn1185
B9H01_RS00255	Bifunctional phosphoribosylaminoimi dazolecarboxamide formyltransferase/inosine monophosphate cyclohydrolase	Tn955
B9H01_RS00275	Hypothetical protein	Tn12
B9H01_RS00315	Membrane protein	Tn1152
B9H01_RS00690	Replication initiation factor domain-containing protein	Tn1374
B9H01_RS00710	Hypothetical protein	Tn97
B9H01_RS00770	Tyrosine--trna ligase	Tn832
B9H01_RS00800	Competence protein (CglA)	Tn1523
B9H01_RS00820	Type II secretion pathway, pseudopilin (PulG)	Tn1761
B9H01_RS00860	DUF4651 domain-containing protein	Tn1597
B9H01_RS00940	Merr family transcriptional regulator	Tn323
B9H01_RS00985	Sugar ABC transporter permease	Tn1
B9H01_RS01045	Glycoside hydrolase family 1 protein	Tn392
B9H01_RS01070	Transketolase	Tn883
B9H01_RS01110	Chemotaxis protein	Tn84
B9H01_RS01125	Formate C-acetyltransferase	Tn1862

Locus Tag	Protein Name	Tn Mutants
B9H01_RS01185	ABC transporter permease	Tn414
B9H01_RS01200	Glucosamine-6-phosphate isomerase	Tn1228
B9H01_RS01205	Copper-exporting atpase	Tn1344
B9H01_RS01295	Transcriptional regulator	Tn485
B9H01_RS01305	Hypothetical protein	Tn472
B9H01_RS01320	Mechanosensitive ion channel family protein	Tn892
B9H01_RS01335	NADP-specific glutamate dehydrogenase	Tn1421
B9H01_RS01360	Membrane protein	Tn1006
B9H01_RS01440	Methyl-accepting chemotaxis protein	Tn1848
B9H01_RS01500	MATE family efflux transporter	Tn652
B9H01_RS01550	Heat-inducible transcriptional repressor (HrcA)	Tn548
B9H01_RS01665	NADH: flavin oxidoreductase	Tn748
B9H01_RS01695	Glycosyl transferase	Tn1654
B9H01_RS01790	Pyridoxal phosphate-dependent aminotransferase	Tn1512
B9H01_RS01925	Hypothetical protein	Tn830
B9H01_RS01960	Peptide deformylase	Tn1100
B9H01_RS01985	Endonuclease	Tn1182
B9H01_RS01990	PTS glucose transporter subunit IIABC	Tn715
B9H01_RS01995	DUF2812 domain-containing protein	Tn1014
B9H01_RS02050	Ribosomal RNA small subunit methyltransferase G	Tn181
B9H01_RS02250	23S rrna (uracil(1939)-C(5))-methyltransferase rlmd	Tn1137
B9H01_RS02275	Gntr family transcriptional regulator	Tn1332
B9H01_RS02285	PTS system mannose/fructose/N-acetylgalactosamine-transporter subunit IIB	Tn1680

Locus Tag	Protein Name	Tn Mutants
B9H01_RS02345	DEAD/DEAH box helicase	Tn858
B9H01_RS02360	Hypothetical protein	Tn862
B9H01_RS02395	Ribonuclease G	Tn1888
B9H01_RS02485	ATP-dependent Clp protease ATP-binding subunit	Tn394
B9H01_RS02495	Amino acid ABC transporter permease	Tn735
B9H01_RS02520	Signal peptidase I	Tn128
B9H01_RS02525	Hypothetical protein	Tn147
B9H01_RS02545	Merr family transcriptional regulator	Tn1034
B9H01_RS02580	Bioy family transporter	Tn636
B9H01_RS02585	Cyclase	Tn1467
B9H01_RS02610	N5,N10-methylene tetrahydromethanopterin reductase	Tn1113
B9H01_RS02615	Hypothetical protein	Tn1055
B9H01_RS02720	Hypothetical protein	Tn1907
B9H01_RS02750	Igm protease	Tn1443
B9H01_RS02830	PLP-dependent aminotransferase family protein	Tn110
B9H01_RS03095	MATE family efflux transporter	Tn1693
B9H01_RS03140	Carbamate kinase	Tn220
B9H01_RS03240	Iron ABC transporter permease	Tn1210
B9H01_RS03295	Yitt family protein	Tn1091
B9H01_RS03355	Hypothetical protein	Tn1717
B9H01_RS03360	Amino acid permease	Tn674
B9H01_RS03370	DUF975 domain-containing protein	Tn438
B9H01_RS03505	MULTISPECIES: glycyl-radical enzyme activating protein	Tn1543
B9H01_RS03575	Glycerol kinase	Tn725
B9H01_RS03585	Aquaporin family protein	Tn259
B9H01_RS03620	HAD family hydrolase	Tn847
B9H01_RS03635	Thiamine phosphate synthase	Tn1602

Locus Tag	Protein Name	Tn Mutants
B9H01_RS03640	MULTISPECIES: Crp/Fnr family transcriptional regulator	Tn782
B9H01_RS03815	Hypothetical protein	Tn416
B9H01_RS03900	SAM-dependent methyltransferase	Tn1466
B9H01_RS03935	Deacetylase	Tn1143
B9H01_RS04005	Guanosine monophosphate reductase	Tn286
B9H01_RS04025	Histidinol-phosphate aminotransferase	Tn1613
B9H01_RS04080	Hypothetical protein	Tn57
B9H01_RS04085	Hypothetical protein	Tn184
B9H01_RS04210	Atpase AAA	Tn473
B9H01_RS04255	Membrane protein	Tn1606
B9H01_RS04285	Gntr family transcriptional regulator	Tn900
B9H01_RS04295	Signal recognition particle protein	Tn1710
B9H01_RS04310	Adenylyl-sulfate kinase	Tn1447
B9H01_RS04315	Abortive phage infection protein	Tn1261
B9H01_RS04455	Membrane protein	Tn474
B9H01_RS04460	ABC transporter ATP-binding protein	Tn1149
B9H01_RS04470	Pqqd family protein	Tn1063
B9H01_RS04500	MULTISPECIES: transposase	Tn641
B9H01_RS04505	MULTISPECIES: excisionase	Tn1778
B9H01_RS04520	MULTISPECIES: sigma-70 family RNA polymerase sigma factor	Tn840
B9H01_RS04530	MULTISPECIES: conjugal transfer protein	Tn1231
B9H01_RS04550	MULTISPECIES: peptidase P60	Tn378
B9H01_RS04585	MULTISPECIES: hypothetical protein	Tn1244
B9H01_RS04595	MULTISPECIES: DUF961 domain-containing protein	Tn1166

Locus Tag	Protein Name	Tn Mutants
B9H01_RS04665	ABC transporter permease	Tn1224
B9H01_RS04685	MULTISPECIES: hypothetical protein	Tn1651
B9H01_RS04690	MULTISPECIES: recombinase	Tn139
B9H01_RS04720	MULTISPECIES: adenine phosphoribosyltransferase	Tn1908
B9H01_RS04735	Type A2 lantipeptide	Tn1827
B9H01_RS04770	Transcriptional regulator	Tn616
B9H01_RS04800	MULTISPECIES: hypothetical protein	Tn360
B9H01_RS04820	MULTISPECIES: hypothetical protein	Tn1037
B9H01_RS04835	DNA (cytosine-5-)-methyltransferase	Tn1758
B9H01_RS04890	DUF1836 domain-containing protein	Tn28
B9H01_RS04905	Ribonuclease HII	Tn1547
B9H01_RS04925	5\'-nucleotidase	Tn1555
B9H01_RS04940	Glycosyltransferase	Tn642
B9H01_RS04970	Lysr family transcriptional regulator	Tn1625
B9H01_RS04980	Glucose-1-phosphate adenylyltransferase subunit (GlgD)	Tn447
B9H01_RS04985	Glycogen synthase (GlgA)	Tn519
B9H01_RS05025	DUF2971 domain-containing protein	Tn1455
B9H01_RS05030	Excinuclease ABC subunit B	Tn925
B9H01_RS05115	Hypothetical protein	Tn418
B9H01_RS05165	Paai family thioesterase	Tn243
B9H01_RS05200	Adenylate cyclase	Tn1539
B9H01_RS05240	Class A sortase	Tn1825
B9H01_RS05340	Hypothetical protein	Tn388
B9H01_RS05380	SAM-dependent methyltransferase	Tn513
B9H01_RS05385	Myo-inositol-1(or 4)-monophosphatase	Tn1736
B9H01_RS05390	MULTISPECIES: hypothetical protein	Tn1360

Locus Tag	Protein Name	Tn Mutants
B9H01_RS05490	Padr family transcriptional regulator	Tn608
B9H01_RS05580	DUF2087 domain-containing protein	Tn1039
B9H01_RS05630	2-dehydro-3-deoxy-phosphogluconate aldolase	Tn596
B9H01_RS05635	Fadr family transcriptional regulator	Tn891
B9H01_RS05655	DNA/RNA non-specific endonuclease	Tn48
B9H01_RS05665	UDP-N-acetylglucosamine 1-carboxyvinyltransferase	Tn140
B9H01_RS05795	DUF1846 family protein	Tn163
B9H01_RS05820	Aconitate hydratase (AcnA)	Tn452
B9H01_RS05855	Hyaluronidase	Tn1803
B9H01_RS05865	Hyaluronidase	Tn707
B9H01_RS05890	PTS system mannose/fructose/N-acetylgalactosamine-transporter subunit IIB	Tn1588
B9H01_RS05895	Glucuronyl hydrolase	Tn694
B9H01_RS05910	Sugar kinase	Tn190
B9H01_RS05915	MULTISPECIES: hypothetical protein	Tn1896
B9H01_RS05940	Hypothetical protein	Tn1010
B9H01_RS06000	O-methyltransferase	Tn1789
B9H01_RS06035	3-deoxy-7-phosphoheptulonate synthase	Tn1834
B9H01_RS06050	3-dehydroquinate synthase	Tn1791
B9H01_RS06055	Hypothetical protein	Tn1738
B9H01_RS06060	ABC transporter ATP-binding protein	Tn619
B9H01_RS06100	Prephenate dehydrogenase	Tn1419
B9H01_RS06285	ZIP family metal transporter	Tn1528
B9H01_RS06420	Glutathione-dependent disulfide-bond oxidoreductase	Tn617

Locus Tag	Protein Name	Tn Mutants
B9H01_RS06470	Sugar ABC transporter permease	Tn237
B9H01_RS06485	Glyoxalase/bleomycin resistance/extradiol dioxygenase family protein	Tn175
B9H01_RS06490	DUF2500 domain-containing protein	Tn1496
B9H01_RS06660	ABC transporter permease	Tn437
B9H01_RS06680	Copper-translocating P-type atpase	Tn14
B9H01_RS06685	Peptidase C69	Tn587
B9H01_RS06760	PTS glucose transporter subunit IIBC	Tn1235
B9H01_RS06770	Thiol-activated toxin suilysin	Tn1806
B9H01_RS06775	Haloacid dehalogenase	Tn1400
B9H01_RS06880	TVP38/TMEM64 family protein	Tn1187
B9H01_RS06955	MULTISPECIES: vanz family protein	Tn1461
B9H01_RS07005	Type I restriction endonuclease subunit R	Tn390
B9H01_RS07055	Serine/threonine transporter (SstT)	Tn912
B9H01_RS07080	ABC transporter ATP-binding protein	Tn1751
B9H01_RS07175	ABC transporter permease	Tn1408
B9H01_RS07300	Site-specific integrase	Tn1916
B9H01_RS07335	Hypothetical protein	Tn1782
B9H01_RS07385	Site-specific integrase	Tn1712
B9H01_RS07415	Chorismate mutase	Tn377
B9H01_RS07420	Voltage-gated chloride channel protein	Tn722
B9H01_RS07445	Hypothetical protein	Tn806
B9H01_RS07450	Hypothetical protein	Tn800
B9H01_RS07470	Branched-chain amino acid ABC transporter permease	Tn433
B9H01_RS07500	Sucrose phosphorylase	Tn1611

Locus Tag	Protein Name	Tn Mutants
B9H01_RS07530	Pyridoxal phosphate-dependent aminotransferase	Tn676
B9H01_RS07535	Cystathionine gamma-synthase	Tn310
B9H01_RS07540	Hypothetical protein	Tn1478
B9H01_RS07550	Hypothetical protein	Tn884
B9H01_RS07610	Histidine triad protein	Tn121
B9H01_RS07670	Hypothetical protein	Tn777
B9H01_RS07710	DUF2974 domain-containing protein	Tn836
B9H01_RS07740	Hypothetical protein	Tn1082
B9H01_RS07810	Yihy/virulence factor brkb family protein	Tn1853
B9H01_RS07850	Peptidase M42	Tn51
B9H01_RS08090	23S rrna (adenine(2503)-C(2))-methyltransferase (RlmN)	Tn182
B9H01_RS08185	Trna(cytidine(34)-2\'-O) methyltransferase	Tn441
B9H01_RS08190	Hypothetical protein	Tn1865
B9H01_RS08275	Membrane protein insertase (YidC)	Tn1900
B9H01_RS08520	Hypothetical protein	Tn1336
B9H01_RS08555	Methylenetetrahydrofolat e reductase	Tn1535
B9H01_RS08565	Cof-type HAD-IIB family hydrolase	Tn365
B9H01_RS08605	DEAD/DEAH box helicase	Tn1180
B9H01_RS08615	Site-specific integrase	Tn512
B9H01_RS08635	N-acetyltransferase	Tn215
B9H01_RS08750	PTS beta-glucoside transporter subunit IIBCA	Tn346
B9H01_RS08760	Laci family transcriptional regulator	Tn1636
B9H01_RS08785	Excinuclease ABC subunit (UvrA)	Tn1510
B9H01_RS08860	Lipoprotein	Tn1264
B9H01_RS08930	Methyl-accepting chemotaxis protein	Tn1345
B9H01_RS09075	Sugar translocase	Tn1633
B9H01_RS09155	ABC transporter ATP-binding protein	Tn1813

Locus Tag	Protein Name	Tn Mutants
B9H01_RS09185	Hypothetical protein	Tn1537
B9H01_RS09190	Sugar ABC transporter substrate-binding protein	Tn1629
B9H01_RS09225	Alpha-1,2-mannosidase	Tn1836
B9H01_RS09230	Endo-beta-N-acetylglucosaminidase	Tn1069
B9H01_RS09270	GNAT family acetyltransferase	Tn143
B9H01_RS09350	3-isopropylmalate dehydratase small subunit	Tn267
B9H01_RS09355	3-isopropylmalate dehydratase large subunit	Tn152
B9H01_RS09405	Dihydroxyacetone kinase subunit (DhaK)	Tn767
B9H01_RS09460	Nuclease	Tn1621
B9H01_RS09520	Adenylate kinase	Tn209
B9H01_RS09680	Tatd family deoxyribonuclease	Tn631
B9H01_RS09705	Argininosuccinate lyase	Tn277
B9H01_RS09740	Amino acid permease	Tn117
B9H01_RS09845	Serine/threonine protein phosphatase	Tn1556
B9H01_RS09920	Pullulanase	Tn76
B9H01_RS09985	6-phospho-beta-glucosidase	Tn1875
B9H01_RS10115	Carboxypeptidase regulatory-like domain-containing protein	Tn1102
B9H01_RS10125	Membrane protein	Tn585
B9H01_RS10185	Metallophosphatase	Tn865
B9H01_RS10245	Hypothetical protein	Tn424
B9H01_RS10260	Sugar ABC transporter permease	Tn1851
B9H01_RS10280	Type I pullulanase	Tn39
B9H01_RS10315	Glycosyl hydrolase	Tn376
B9H01_RS10335	ATP-binding protein	Tn973
B9H01_RS10340	Phosphoesterase	Tn1279
